# Pyviko: an automated Python tool to design gene knockouts in complex viruses with overlapping genes

**DOI:** 10.1186/s12866-016-0920-3

**Published:** 2017-01-07

**Authors:** Louis J. Taylor, Klaus Strebel

**Affiliations:** 1 Viral Biochemistry Section, Laboratory of Molecular Microbiology, National Institute of Allergy and Infectious Diseases, National Institutes of Health, Bethesda, MD USA; 2 Cell and Molecular Biology Graduate Group, Perelman School of Medicine, University of Pennsylvania Philadelphia, Pennsylvania, USA

**Keywords:** Virus, Knockout virus, Mutation, Overprinting, Bioinformatics, Cloning, Viral mutant, Knockout, Pyviko

## Abstract

**Background:**

Gene knockouts are a common tool used to study gene function in various organisms. However, designing gene knockouts is complicated in viruses, which frequently contain sequences that code for multiple overlapping genes. Designing mutants that can be traced by the creation of new or elimination of existing restriction sites further compounds the difficulty in experimental design of knockouts of overlapping genes. While software is available to rapidly identify restriction sites in a given nucleotide sequence, no existing software addresses experimental design of mutations involving multiple overlapping amino acid sequences in generating gene knockouts.

**Results:**

Pyviko performed well on a test set of over 240,000 gene pairs collected from viral genomes deposited in the National Center for Biotechnology Information Nucleotide database, identifying a point mutation which added a premature stop codon within the first 20 codons of the target gene in 93.2% of all tested gene-overprinted gene pairs. This shows that Pyviko can be used successfully in a wide variety of contexts to facilitate the molecular cloning and study of viral overprinted genes.

**Conclusions:**

Pyviko is an extensible and intuitive Python tool for designing knockouts of overlapping genes. Freely available as both a Python package and a web-based interface (http://louiejtaylor.github.io/pyViKO/), Pyviko simplifies the experimental design of gene knockouts in complex viruses with overlapping genes.

**Electronic supplementary material:**

The online version of this article (doi:10.1186/s12866-016-0920-3) contains supplementary material, which is available to authorized users.

## Background

**Fig. 1:**
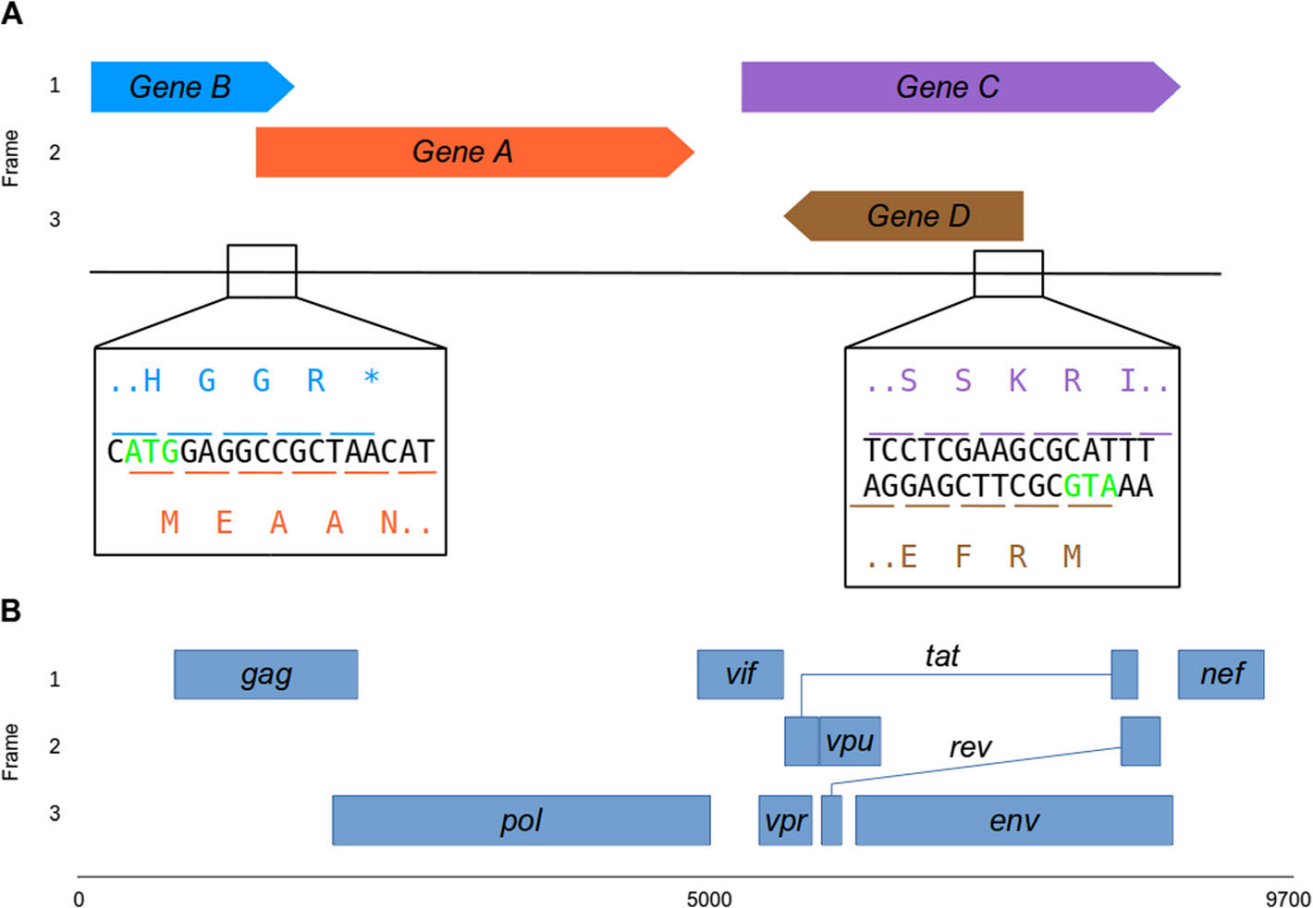
Examples of gene overprinting in viruses. **a** Outline showing different types of gene overprinting and associated nucleotide and amino acid sequences. *Gene B* overprints into *gene A* in the same direction but a different frame. Genes *C* and *D* showcase overprinting in different frames and directions. Start codons in nucleotide sequences are shown in green. **b** Genome of human immunodeficiency virus type I [7] with annotated genes. *tat* and *rev* splicing is indicated by a solid line

Gene knockouts are an important tool used to study gene function in viruses [1], bacteria [2], and other organisms, including model organisms such as mice [3]. Although the principle of removing a gene in an attempt to discern its cellular role is not new, the recent development of CRISPR/Cas9 as a tool for knocking out genes in vivo has revolutionized the field of genome editing and underscores the importance of using knockouts as a tool to study gene function [4]. A common experimental approach to knocking out a gene is to simply excise the gene of interest from the target organism’s genome. Viruses, however, often contain DNA sequences that code for multiple protein products in separate reading frames, called overprinted genes (Fig. 1a) [5, 6]. For, example, human immunodeficiency virus type 1 (HIV-1) contains 8 instances of gene overprinting [7], as shown in Fig. 1b. This phenomenon is widespread among different viral families [5] and precludes the excision strategy of knocking out a viral gene.

An alternate approach to excising genes is to mutate a sequence in order to insert a premature stop codon in the target gene, which results in a non-functional truncation of the final translated protein. Throughout this work, “target gene” refers to the gene we wish to mutate in order to insert a premature stop codon. In Fig. 1a, *gene A* is the target (overprinted) gene, and *gene b* is the overlapping gene. To ensure that this truncation retains no function of the wild-type gene, the stop codon should occur as early in the gene sequence as possible. However, the amino acid sequence of the overlapping gene must be preserved in order to experimentally discriminate between changes in phenotype due to changes in the overlapping and target genes. Such mutations are possible due to the degeneracy of the genetic code—several amino acids can be coded for by more than one nucleotide sequence [1]. However, designing such mutants by hand is slow and non-trivial, especially when mutating many clones or strains of viruses. Additionally, changes in restriction enzyme recognition sites that can be used to trace the newly introduced mutations are important experimentally in planning molecular cloning and mutagenesis protocols [8].

Restriction enzymes are a class of bacterial endonucleases that specifically cleave DNA at a 4–8 nucleotide recognition sequence. Mutagenesis protocols are frequently designed to add or remove a restriction site as tracers, so that resulting constructs can be analyzed inexpensively for the presence of the desired mutation without having to sequence multiple clones [8]. Given the diversity and number of restriction enzymes commercially available [9], searching for potential restriction enzyme recognition sites in a given DNA sequence by hand is unfeasible. Methods for rapid searching of nucleotide sequences for restriction sites have been previously published [10] and are not discussed here. In this work, we introduce Pyviko—a tool which automates the process of designing knockout viruses while taking into account changes in restriction enzyme recognition sites and the integrity of the overlapping gene.

## Implementation

Pyviko was implemented in Python 2.7. The Python regex module [11] is optional and can be installed to augment the base functionality of Pyviko, but is not required to use the software. Source code is freely available [12] under the MIT license and is thoroughly unit tested prior to each release. Releases are available from the Python Package Index [13]. A client-side web interface in JavaScript is available for making single-gene knockouts without installing Pyviko [14]. Extensive documentation for Pyviko is available online [15] and as comments in the source code. Online documentation is build directly from comments in the source using pdoc [16] and is available without installing Pyviko. Bug reports should be submitted on the project’s GitHub page [12].

The basic functionality of Pyviko is divided into three modules: core, restriction, and mutation. Functions for basic nucleic acid sequence manipulation, including reading from and writing to Fast-All (FASTA) files, are included within the core module. The restriction module contains functions to analyze sequences for restriction sites and find changes in restriction sites that result from sequence changes. The mutation module includes functions to find favorable mutations as well as the Mutant and OverGene classes. The Mutant class brings together the three modules and identifies favorable knockout mutants in sequences of interest.

## Results

### Use in interactive and stand-alone scripts

**Fig. 2:**
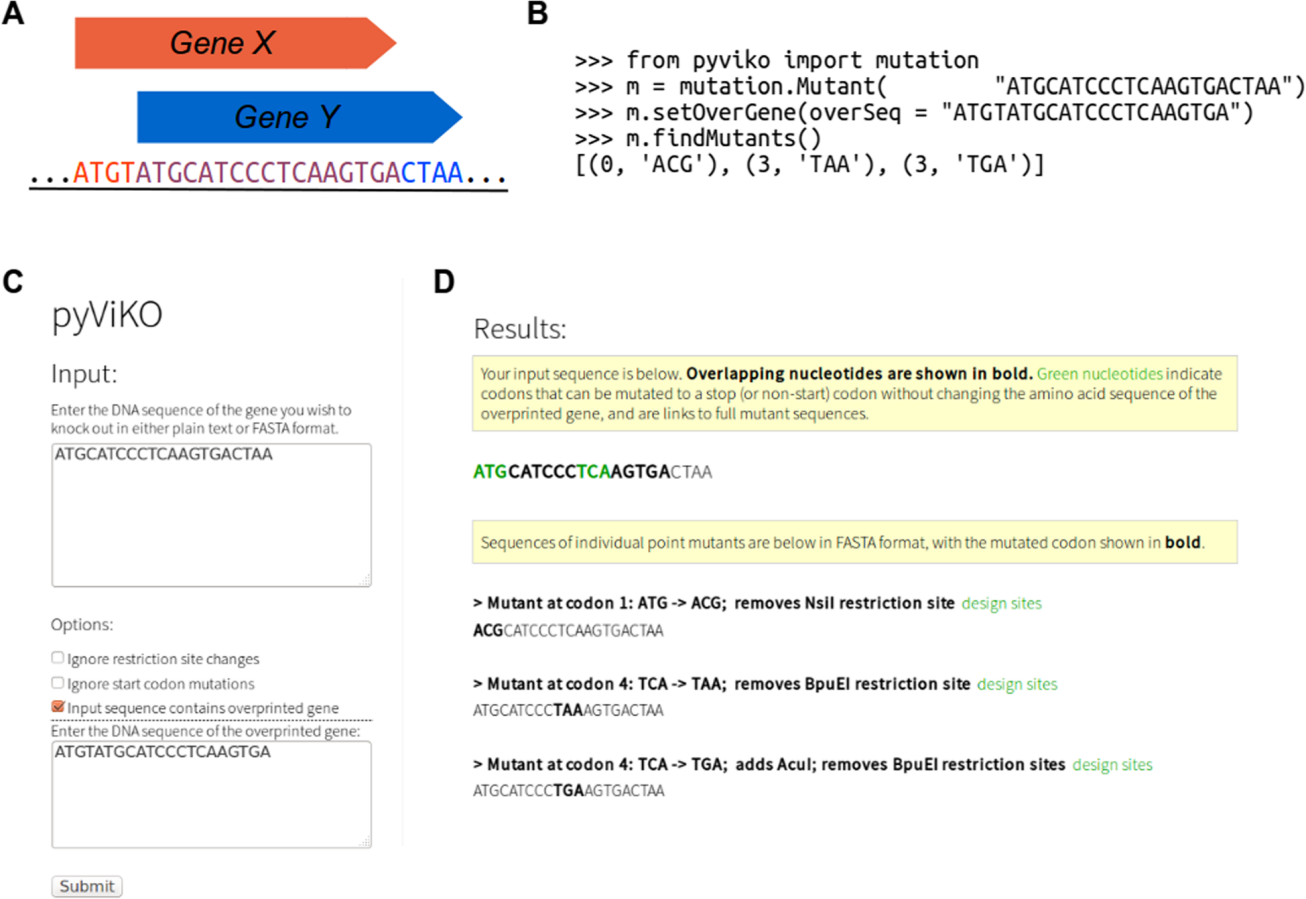
Usage of Pyviko in stand-alone scripts and web interface. **a** Schematic of sample target (*Y*) and overlapping (*X*) genes used in code examples. **b** Minimal Python commands for generating gene knockouts. In this interactive example, “>>>” denotes input into a Python interpreter and output is displayed directly below the input on an unindented line. **c** Pyviko input interface [14] including sample gene sequences. **d** Results of analysis for input shown in (**c**)

Pyviko was created to streamline the process of planning viral mutageneses. Each module is designed to be intuitive and extensible to facilitate its use in a variety of applications. Figure 2a–b shows the generation of knockouts from a single target and overlapping gene pair in an interactive fashion in a Python interpreter. Using the sequences of an input gene and its overprinted counterpart, the overlapping sequence is automatically detected and all possible knockouts matching the input parameters are displayed. Thus, in the example shown in Fig. 2a, the target gene can be knocked out by mutating the initiation codon (index 0) to ACG without changing the coding capacity of the overlapping gene (TAT and TAC both code for tyrosine). Alternatively, the TCA (serine) codon at index 3 of the target gene can be mutated to a stop codon (TGA or TAA) without changing the coding sequence of the overlapping gene (CTC, CTA and CTG all encode leucine).

To reach a wider, non-computational audience, this single-gene pair knockout script has also been implemented as a graphical interface in JavaScript [14]. This interface supports generation of knockouts for a gene or pair of genes under various parameters, including requirements for start codon mutants or restriction site changes. Figure 2c shows the input interface and 2D shows the output of the analysis for the gene pair shown in Fig. 2a. The full mutant sequence for each mutation is shown together with a link to an interface to design further restriction site changes for a particular point mutant.

For experiments requiring the generation of many mutants, Pyviko provides a rapid, high-throughput approach to viral mutagenesis. Additionally, Pyviko supports reading from and writing to files in the universal FASTA format to maximize inter-application compatibility. Additional file 1 contains a Python script showcasing the ability of Pyviko to generate mutants in a batched fashion, taking input from FASTA files and writing mutagenesis results to FASTA. Additional file 1 also shows many of the options available when searching sequences, including filtering for mutants that add or remove a restriction site and including or excluding mutants that perturb the start codon. Mutating a gene’s start codon is another feasible option to prevent production of a specific protein. Some viruses, however, have been shown to use alternate start codons [17]. Care should be taken to biologically validate knockouts made using this strategy.

### Integration with existing tools and extensibility

Recent trends toward open-source software in bioinformatics have led to the development of a variety of new Python tools for analyzing nucleotide sequences, including Biopython [10] and Pydna [18]. While Biopython and Pydna both include functions for restriction site identification, neither is designed to analyze restriction site changes as a result of nucleotide sequence mutations. Pyviko is designed to solve the problem of mutating overprinted genes, which are most common in viruses but do occur in other organisms, including bacteria [19], mice, and humans [20]. Unlike other software, Pyviko is optimized to analyze multiple overlapping sequences concurrently, examining changes in translated amino acid sequence and restriction sites resulting from changes in nucleotide sequence.

While Pyviko is not dependent on existing software for its basic nucleic acid manipulation and restriction site identification functionalities, it is not designed to supersede other general nucleotide sequence analysis programs. Rather, Pyviko can be used in conjunction with software like Pydna and Biopython to complement Pyviko’s approach to viral mutagenesis. Additional file 2 contains the script used to collect sequences from GenBank for the large-scale analysis discussed in the following section. This script leverages Biopython’s Entrez module to retrieve viral genomes to be analyzed by Pyviko. Additional file 3 is a Python script that retrieves a viral genome sequence (HIV-1 NL4-3 [21]) from the NCBI Nucleotide database [22] via Biopython, generates a knockout for a target overprinted gene (*vpr*) with Pyviko, then uses Pydna to design primers for molecular cloning.

Although Pyviko was designed to generate knockouts of viral overprinted genes, the software is generally applicable to any mutagenesis of overprinted genes. Additional file 4 is a Python script containing a variety of examples of mutagenesis design involving overprinted genes, including: mutagenesis of hydrophobic to non-hydrophobic amino acid residues in the target gene, identification of all mutations in the overprinted region that do not change the polypeptide sequence of the overlapped gene, and generating a sequence that scrambles the amino acids of a target gene without changing the amino acids of the overlapping gene.

### Large-scale functional testing

To validate the approach of Pyviko in knocking out overprinted viral genes, we performed a large-scale analysis of annotated viral genomes deposited in the National Center for Biotechnology Information (NCBI) Nucleotide database. Using the script included as Additional file 2, we collected 48,770 sequences annotated as complete viral genomes from the NCBI Nucleotide database. From these genomes, 248,777 pairs of overprinted gene pairs were identified and analyzed by Pyviko. Note that individual genes may appear in more than one pair as a gene may overlap with more than one other gene (e.g. *vif* gene in Fig. 1b).

Pyviko was able to identify point mutants that added a premature stop codon in the target gene without changing the amino acid sequence of the overlapped gene in 96.5% of all genes analyzed. Many of these mutations added or removed a restriction enzyme recognition site [9], and 96.2% of all target genes analyzed could be knocked out as above with the additional constraint of a restriction site change. However, the location of the premature stop codon is important for the efficacy of the knockout—a “premature” stop codon close to the end of the target gene could still result in a gene product with some level of function. Thus, we decided to further judge the efficacy of Pyviko knockouts by examining the first possible premature stop codon in each target gene identified by Pyviko.

**Fig. 3:**
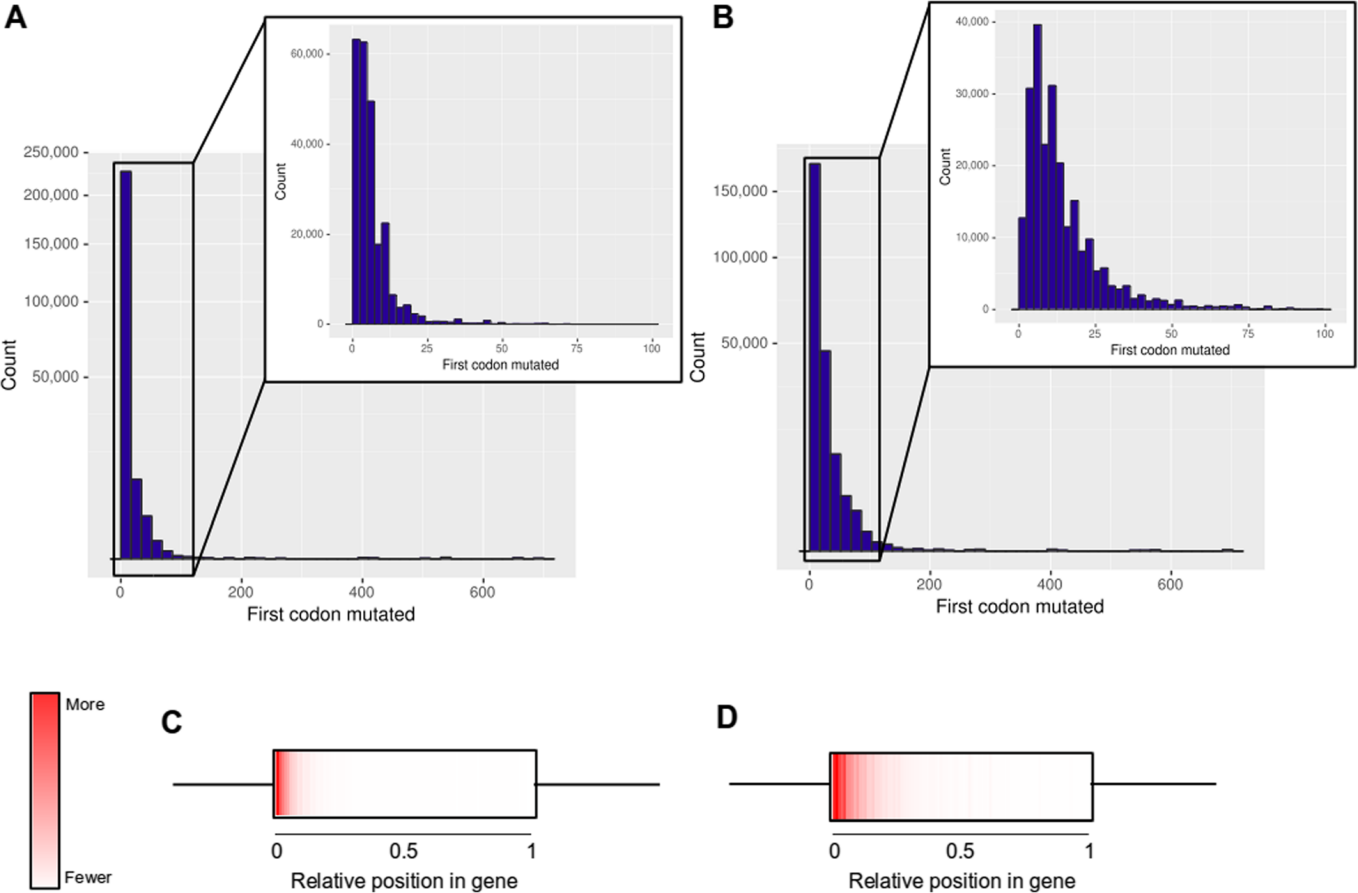
Large-scale analysis of viral overprinted genes from the NCBI Nucleotide database. **a** and **b** Show counts of the first position at which a directed point mutation can insert a premature stop codon in the overprinted gene without changing the amino acid sequence of the overlapped gene in each gene pair surveyed. Full-size graphs show mutations over the full length of all genes surveyed with a log_2_ scale to ensure visibility of bins with low counts. Insets show the first 100 codons of each gene with a linear y axis. **a** Shows counts without requiring a restriction site change and (**b**) shows counts with a required restriction site change. **c** and **d** Show positions of the first stop codon mutants from (**a**) and (**b**) expressed as the ratio of the position of the first codon relative to the total length of the gene in codons. **c**, as in (**a**), shows counts without requiring a restriction site change and (**d**), as in (**b**), shows counts with a required restriction site change

To quantify the effectiveness of Pyviko knockouts, we calculated the percentage of stop codons that could be added within the first 20 codons of the target gene. While there is evidence that polypeptides shorter than 20 amino acids could have intracellular functions [23], it is highly unlikely that a protein truncated to 20 amino acids or less would retain its original function. Pyviko identified potential premature stop codons within the first 20 codons in 93.2% of all target genes (Fig. 3a). Requiring a restriction site change, Pyviko identified a premature stop codon within the first 20 codons in 76.4% of target genes (Fig. 3b). These data show that, while requiring a restriction site change does not greatly change the percentage of target genes that can be knocked out, the distribution of first premature stop codons is much wider when restriction site changes are required (Fig. 3a–d). It is also possible that the relative truncated length of a knockout is important—for example, a truncated gene coding for 20 amino acids is 20% of the final polypeptide length of a 100 amino acid protein, but only 2% of a 1000 amino acid protein. However, a heat map of first-codon knockouts along a unit gene show that over 90% of knockouts are within the first 10% of the coding sequence both without (Fig. 3c) and with (Fig. 3d) restriction site change constraints. This is consistent with the conclusion that the vast majority of Pyviko-designed gene knockouts will not result in a functional protein product.

## Conclusions

In this work, we introduce Pyviko, an intuitive and extensible Python tool for designing viral knockouts. While the software is platform-independent and does not require any external modules other than Python itself, Pyviko can also be used with existing Python tools such as Biopython and Pydna to extend its base functionality as shown in the example scripts. Pyviko performed well on a test set of over 240,000 gene pairs collected from viral genomes deposited in the NCBI Nucleotide database, identifying a point mutation that could be inserted within the first 20 codons of the target gene in 93.2% of all tested gene-overprinted gene pairs. This shows that Pyviko can be used successfully in a wide variety of contexts to facilitate the molecular cloning and study of viral overprinted genes. The complete source code and quick-start guide are included as Additional files 5 and 6, respectively.

## Availability and requirements

Project name: Pyviko

Project home pages: https://github.com/louiejtaylor/pyViKO, https://pypi.python.org/pypi/pyviko

Operating system(s): Platform independent

Programming language: Python

Other requirements: Python 2.7 or higher

License: MIT license

## Additional files

Additional file 1: A Python script showcasing the ability of Pyviko to generate mutants in a batched fashion, taking input from FASTA files and writing mutagenesis results to FASTA. Additional file 1 also shows many of the options available when searching sequences, including filtering for mutants that add or remove a restriction site and including or excluding mutants that perturb the start codon. (PY 787 bytes)
Additional file 2: The Python script used to collect sequences from GenBank for the large-scale analysis in Fig. 3. This script leverages Biopython’s Entrez module to retrieve viral genomes to be analyzed by Pyviko. (PY 4 kb)
Additional file 3: A Python script that retrieves a viral genome sequence (HIV-1 NL4-3) from the NCBI Nucleotide database via Biopython, generates a knockout for a target overprinted gene (*vpr* is overprinted by *vif*) with Pyviko, then uses Pydna to design primers for molecular cloning. (PY 1 kb)
Additional file 4: A Python script containing a variety of examples of mutagenesis design involving overprinted genes: mutagenesis of hydrophobic to non-hydrophobic amino acid residues in the target gene, identification of all mutations in the overprinted region that do not change the polypeptide sequence of the overlapped gene, and generating a sequence that scrambles the amino acids of the target gene without changing the amino acids of the overlapped gene. (PY 4 kb)
Additional file 5: The Pyviko Web User Interface Quick Start Guide, also available at [24]. This document explains the use of the Pyviko web user interface. (PDF 461 kb)
Additional file 6: The Pyviko source code, version 1.0.1.1. Current release available from the Python Package Index [13]. (GZ 7 kb)

## Abbreviations

DNA: Deoxyribonucleic acid
FASTA: Fast-All (file format)
HIV-1: Human immunodeficiency virus type 1
NCBI: National Center for Biotechnology Information
Pyviko: Python viral knockouts
RNA: Ribonucleic acid
