# Supplementary material for: Pyviko: an automated Python tool to design gene knockouts in complex viruses with overlapping genes
Source: BMC Microbiol. 2017 Jan 7;17:12. doi: 10.1186/s12866-016-0920-3 (PMC5219722; doi:10.1186/s12866-016-0920-3)
Supplement: Additional file 5: — The Pyviko Web User Interface Quick Start Guide, also available at [24]. This document explains the use of the Pyviko web user interface. (PDF 461 kb) [file 12866_2016_920_MOESM5_ESM.pdf]

Pyviko Web UI Quick Start Guide

LJ Taylor

Table of Contents

1. Introduction 2
2. Gene knockouts with Pyviko 2
    a. Input formats 2
    b. Results 3
3. Restriction site design with Pyviko 4
    a. Input formats 4
    b. Results 5

1. Introduction

What is Pyviko?

Pyviko stands for Python Viral Knockouts (stylized: pyViKO). Pyviko is a tool for designing molecular cloning protocols in complex viruses or other organisms with overlapping genes. This web version of Pyviko finds mutations in a target gene that insert a premature stop codon without changing the amino acid sequence of the overprinted gene. The online version of Pyviko also includes a restriction site designer, helping to identify co-occuring mutations that also do not change the amino acid sequence of the overlapping gene but either add or remove a restriction enzyme recognition site.

What is an “overprinted gene”?

An overprinted gene is defined as the extension of one gene's open reading frame into the reading frame of a second gene. A single DNA sequence can code for multiple proteins in different reading frames or by reading in different directions. For more information, see the Wikipedia article on reading frames or this (open access) paper on the origins of overprinted genes.

2. Gene knockouts with Pyviko

The web interface for Pyviko can be found at http://louiejtaylor.github.io/pyViKO/. For the examples below, we will design knockouts of a gene from Human Immunodeficiency Virus 1 (HIV-1), Vpr, which is overprinted into by another HIV-1 gene, Vpx. cDNA sequences for Vpr and Vpx are below:

```
>Vpr
atggaacaagccccagaagaccaagggccacagagggagccatacaatgaatggacactagagcttttagaggaacttaagag
tgaagctgttagacattttcctaggatatggctccataacttaggacaacatatctatgaaacttacggggatacttgggcag
gagtggaagccataataagaattctgcaacaactgctgtttatccatttcagaattgggtgtcgacatagcagaataggcgtt
actcgacagaggagagcaagaaatggagccagtagatcctag
```

```
>Vpx
atggaaaacagatggcaggtgatgattgtgtggcaagtagacaggatgaggattaacacatggaaaagattagtaaaacacca
tatgtatatttcaaggaaagctaaggactggttttatagacatcactatgaaagtactaatccaaaaataagttcagaagtac
acatcccactaggggatgctaaattagtaataacaacatattggggtctgcatacaggagaaagagactggcatttgggtcag
ggagtctccatagaatggaggaaaaagagatatagcacacaagtagaccctgacctagcagaccaactaattcatctgcacta
ttttgattgtttttcagaatctgctataagaaataccatattaggacgtatagttagtcctaggtgtgaatatcaagcaggac
ataacaaggtaggatctctacagtacttggcactagcagcattaataaaaccaaaacagataaagccacctttgcctagtgtt
aggaaactgacagaggacagatggaacaagccccagaagaccaagggccacagagggagccatacaatgaatggacactag
```

a. Input formats

Pyviko accepts input in FASTA and plaintext format. Input is case-insensitive and whitespace is ignored.

Instructions:

1. Paste the sequence of the target gene (Vpr) into the first textbox.
2. Choose the options for the type of knockouts you wish to make, then press "submit":
   a. If you do not wish to make restriction site changes, check the "Ignore restriction site changes" box.
   b. If you do not wish to include mutations that perturb the start codon, check the "Ignore start codon mutations" box.
   c. If your gene does not include an overprinted counterpart, uncheck the "Input sequence contains overprinted gene" box. Otherwise, paste the overlapping gene (Vpx) sequence here.

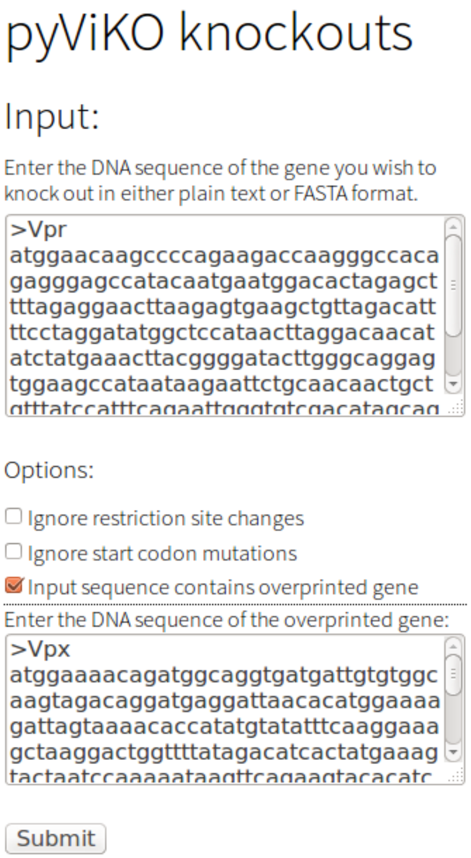

b. Results:

Consensus sequence:

The input sequence is shown first. Nucleotides that overlap in the target and overprinted gene are shown in bold. Green nucleotides are links to full mutant sequences. Each green link is a codon that can be mutated to a stop (or non-start) codon.

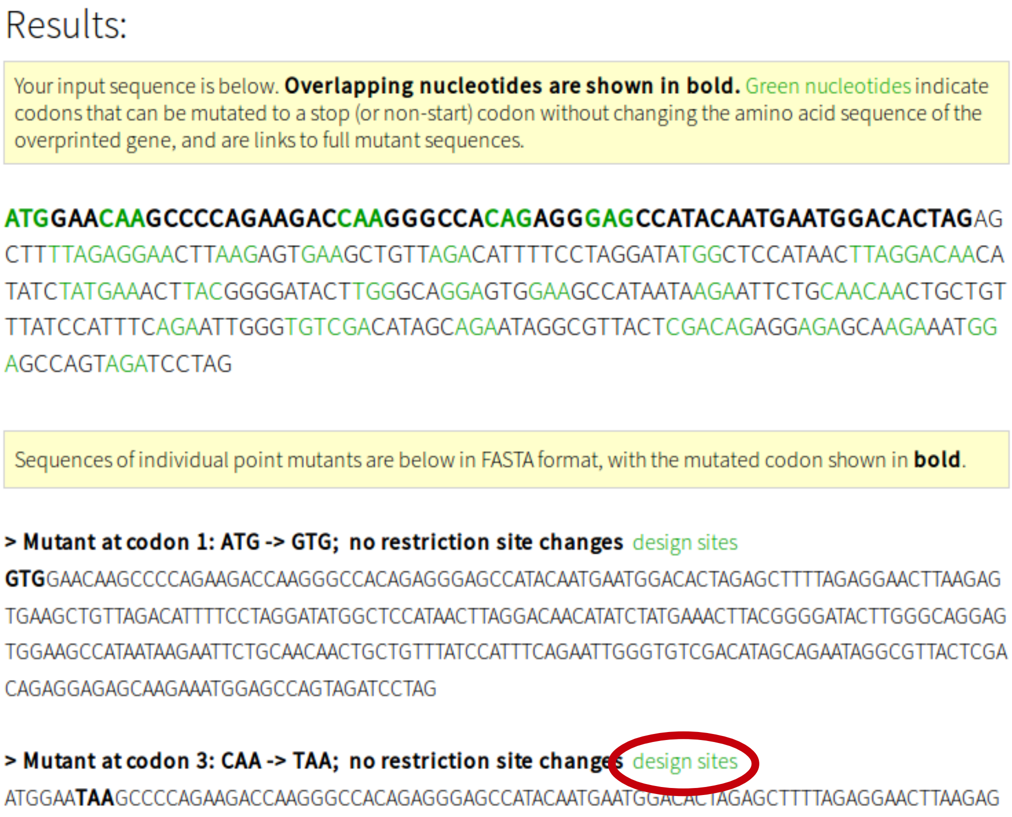

Mutant sequences:

Each sequence below the target consensus sequence is an individual point mutant. A "design sites" link is next to the list (or lack of) restriction site changes which assists in the design of additional restriction site changes in the context of a particular mutant.

3. Restriction site design with Pyviko

The restriction site designer (http://louiejtaylor.github.io/pyViKO/designer.html) accompanies Pyviko's knockout designs by facilitating the addition or deletion of restriction sites for a particular mutant. The screenshots in the below example were generated by clicking the green "design sites" link (circled in red) next to the CAA → TAA mutant at codon 3 of Vpr.

a. Input formats

As before, the restriction designer accepts input in FASTA and plaintext format. Input is case-insensitive and whitespace is ignored.

Instructions:

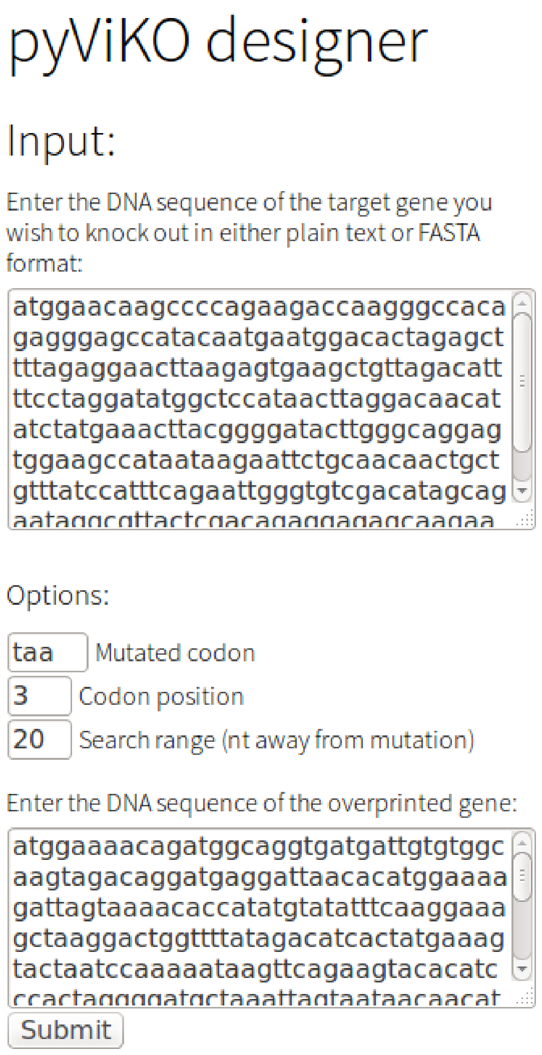

1. The simplest input method is to click the "design sites" link on the knockout design page, as all the relevant information will appear by default.

   OR

1. Paste the sequence of the target gene (Vpr) into the first textbox.
2. Options:
   a. Input the three-nucleotide codon you wish to mutate. Note that the designer does not enforce that the input mutated codon ensures the fidelity of the overprinted gene, as it is assumed that this information is coming from the knockout designer.
   b. Input the position of the mutant codon. The first codon has position 1.
   c. Enter the search range. This is the distance (in both directions) away from the mutated codon to search for restriction site mutations.
3. Enter the sequence of the overlapping gene (Vpx) into the last textbox and press "Submit".

b. Results:

Consensus sequence:

The input sequence is shown first. Nucleotides that overlap in the target and overprinted gene are shown in bold. Green nucleotides are links to full mutant sequences. Each green link is a codon that can be mutated to change a restriction enzyme recognition site without changing the amino acid sequence of the overprinted gene. Note that the consensus sequence already contains the input mutation.

Results of restriction site design:

The input consensus gene sequence is below, including the input mutated codon. **Overlapping nucleotides are shown in bold.** Green nucleotides indicate point mutants that add or remove a restriction site without changing the amino acid sequence of the overprinted gene, and are links to full mutant sequences.

ATGGAAAACAGATGGCAGGTGATGATTGTGTGGCAAGTAGACAGGATGAGGATTAACACATGGAAAAGATT
AGTAAAACACCATATGTATATTTCAAGGAAAGCTAAGGACTGGTTTTATAGACATCACTATGAAAGTACTAA
TCCAAAAATAAGTTCAGAAGTACACATCCCACTAGGGGATGCTAAATTAGTAATAACAACATATTGGGGTC
TGCATACAGGAGAAAGAGACTGGCATTTGGGTCAGGGAGTCTCCATAGAATGGAGGAAAAAGAGATATAG
CACACAAGTAGACCCTGACCTAGCAGACCAACTAATTCATCTGCACTATTTTGATTGTTTTTCAGAATCTG
CTATAAGAAATACCATATTAGGACGTATAGTTAGTCCTAGGTGTGAATATCAAGCAGGACATAACAAGGTAG
GATCTCTACAGTACTTGGCACTAGCAGCATTAATAAAACCAAAACAGATAAAGCCACCTTTGCCTAGTGTT
AGGAAACTGACAGAGGACAG**ATGGAATAAGCCCCAGAAGACCAAGGGCCACAGAGGGAGCCATA**
CAATGAATGGACACTAGAGCTTTTAGAGGAACTTAAGAGTGAAGCTGTTAGACATTTTCCTAGGATATG
GCTCCATAACTTAGGACAACATATCTATGAAACTTACGGGGATACTTGGGCAGGAGTGGAAGCCATAATAA
GAATTCTGCAACAACTGCTGTTTATCCATTTCAGAATTGGGTGTCGACATAGCAGAATAGGCGTTACTCG
ACAGAGGAGAGCAAGAAATGGAGCCAGTAGATCCTAG

Mutant sequences:

Each sequence below the full consensus sequence is an individual point mutant. The full consensus (overlapping + target) sequence is shown for each mutant. The nucleotide in bold is the nucleotide that, when mutated, adds or removes the indicated restriction site.

Sequences of individual point mutants are below, with the mutated nucleotide shown in **bold**.

> Mutant at nt 513: G -> A; adds MboII, BbsI restriction sites

ATGGAAAACAGATGGCAGGTGATGATTGTGTGGCAAGTAGACAGGATGAGGATTAACACATGGAAAAGATTAGTAAAACAC
CATATGTATATTTCAAGGAAAGCTAAGGACTGGTTTTATAGACATCACTATGAAAGTACTAATCCAAAAATAAGTTCAGAAGTA
CACATCCCACTAGGGGATGCTAAATTAGTAATAACAACATATTGGGGTCTGCATACAGGAGAAAGAGACTGGCATTTGGGTC
AGGGAGTCTCCATAGAATGGAGGAAAAAGAGATATAGCACACAAGTAGACCCTGACCTAGCAGACCAACTAATTCATCTGCA
CTATTTTGATTGTTTTTCAGAATCTGCTATAAGAAATACCATATTAGGACGTATAGTTAGTCCTAGGTGTGAATATCAAGCAG
GACATAACAAGGTAGGATCTCTACAGTACTTGGCACTAGCAGCATTAATAAAACCAAAACAGATAAAGCCACCTTTGCCTAGT
GTTAGGAAACTGACAGA**A**GACAGATGGAATAAGCCCCAGAAGACCAAGGGCCACAGAGGGAGCCATACAATGAATGGACAC
TAGAGCTTTTAGAGGAACTTAAGAGTGAAGCTGTTAGACATTTTCCTAGGATATGGCTCCATAACTTAGGACAACATATCTAT
GAAACTTACGGGGATACTTGGGCAGGAGTGGAAGCCATAATAAGAATTCTGCAACAACTGCTGTTTATCCATTTCAGAATTG
GGTGTCGACATAGCAGAATAGGCGTTACTCGACAGAGGAGAGCAAGAAATGGAGCCAGTAGATCCTAG

> Mutant at nt 519: A -> G; removes BccI restriction site

ATGGAAAACAGATGGCAGGTGATGATTGTGTGGCAAGTAGACAGGATGAGGATTAACACATGGAAAAGATTAGTAAAACAC
CATATGTATATTTCAAGGAAAGCTAAGGACTGGTTTTATAGACATCACTATGAAAGTACTAATCCAAAAATAAGTTCAGAAGTA
CACATCCCACTAGGGGATGCTAAATTAGTAATAACAACATATTGGGGTCTGCATACAGGAGAAAGAGACTGGCATTTGGGTC
AGGGAGTCTCCATAGAATGGAGGAAAAAGAGATATAGCACACAAGTAGACCCTGACCTAGCAGACCAACTAATTCATCTGCA
CTATTTTGATTGTTTTTCAGAATCTGCTATAAGAAATACCATATTAGGACGTATAGTTAGTCCTAGGTGTGAATATCAAGCAG
GACATAACAAGGTAGGATCTCTACAGTACTTGGCACTAGCAGCATTAATAAAACCAAAACAGATAAAGCCACCTTTGCCTAGT
GTTAGGAAACTGACAGAGGACAG**G**TGGAATAAGCCCCAGAAGACCAAGGGCCACAGAGGGAGCCATACAATGAATGGACA
CTAGAGCTTTTAGAGGAACTTAAGAGTGAAGCTGTTAGACATTTTCCTAGGATATGGCTCCATAACTTAGGACAACATATCTA
TGAAACTTACGGGGATACTTGGGCAGGAGTGGAAGCCATAATAAGAATTCTGCAACAACTGCTGTTTATCCATTTCAGAATT
GGGTGTCGACATAGCAGAATAGGCGTTACTCGACAGAGGAGAGCAAGAAATGGAGCCAGTAGATCCTAG

> Mutant at nt 531: C -> T; adds BspCNI restriction site

ATGGAAAACAGATGGCAGGTGATGATTGTGTGGCAAGTAGACAGGATGAGGATTAACACATGGAAAAGATTAGTAAAACAC
CATATGTATATTTCAAGGAAAGCTAAGGACTGGTTTTATAGACATCACTATGAAAGTACTAATCCAAAAATAAGTTCAGAAGTA
